# Associations between Pathogens in the Upper Respiratory Tract of Young Children: Interplay between Viruses and Bacteria

**DOI:** 10.1371/journal.pone.0047711

**Published:** 2012-10-17

**Authors:** Menno R. van den Bergh, Giske Biesbroek, John W. A. Rossen, Wouter A. A. de Steenhuijsen Piters, Astrid A. T. M. Bosch, Elske J. M. van Gils, Xinhui Wang, Chantal W. B. Boonacker, Reinier H. Veenhoven, Jacob P. Bruin, Debby Bogaert, Elisabeth A. M. Sanders

**Affiliations:** 1 Department of Pediatric Immunology and Infectious Diseases, Wilhelmina Children’s Hospital, University Medical Center Utrecht, Utrecht, The Netherlands; 2 Research Center Linnaeus Institute, Spaarne Hospital, Hoofddorp, The Netherlands; 3 Laboratory of Medical Microbiology and Immunology, St Elisabeth Hospital, Tilburg, The Netherlands; 4 Julius Center for Health Sciences and Primary Care, University Medical Center Utrecht, Utrecht, The Netherlands; 5 Regional Laboratory of Public Health, Haarlem, The Netherlands; The Ohio State Unversity, United States of America

## Abstract

**Background:**

High rates of potentially pathogenic bacteria and respiratory viruses can be detected in the upper respiratory tract of healthy children. Investigating presence of and associations between these pathogens in healthy individuals is still a rather unexplored field of research, but may have implications for interpreting findings during disease.

**Methodology/Principal Findings:**

We selected 986 nasopharyngeal samples from 433 6- to 24-month-old healthy children that had participated in a randomized controlled trial. We determined the presence of 20 common respiratory viruses using real-time PCR. *Streptococcus pneumoniae*, *Haemophilus influenzae*, *Moraxella catarrhalis* and *Staphylococcus aureus* were identified by conventional culture methods. Information on risk factors was obtained by questionnaires. We performed multivariate logistic regression analyses followed by partial correlation analysis to identify the overall pattern of associations. *S. pneumoniae* colonization was positively associated with the presence of *H. influenzae* (adjusted odds ratio 1.60, 95% confidence interval 1.18–2.16), *M. catarrhalis* (1.78, 1.29–2.47), human rhinoviruses (1.63, 1.19–2.22) and enteroviruses (1.97, 1.26–3.10), and negatively associated with *S. aureus* presence (0.59, 0.35–0.98). *H. influenzae* was positively associated with human rhinoviruses (1.63, 1.22–2.18) and respiratory syncytial viruses (2.78, 1.06–7.28). *M. catarrhalis* colonization was positively associated with coronaviruses (1.99, 1.01–3.93) and adenoviruses (3.69, 1.29–10.56), and negatively with *S. aureus* carriage (0.42, 0.25–0.69). We observed a strong positive association between *S. aureus* and influenza viruses (4.87, 1.59–14.89). In addition, human rhinoviruses and enteroviruses were positively correlated (2.40, 1.66–3.47), as were enteroviruses and human bocavirus, WU polyomavirus, parainfluenza viruses, and human parechovirus. A negative association was observed between human rhinoviruses and coronaviruses.

**Conclusions/Significance:**

Our data revealed high viral and bacterial prevalence rates and distinct bacterial-bacterial, viral-bacterial and viral-viral associations in healthy children, hinting towards the complexity and potential dynamics of microbial communities in the upper respiratory tract. This warrants careful consideration when associating microbial presence with specific respiratory diseases.

## Introduction

Koch’s original postulates, designed to link one causative microbe to one specific disease, have been subject to reconsideration since they were formulated in 1884 [1]–[3]. In fact, Koch himself abandoned his first postulate when he discovered that the causative agent of cholera could also be carried asymptomatically [1]. Since then, it is increasingly acknowledged that human diseases, including respiratory tract infections like otitis media and pneumonia, are polymicrobial–resulting from synergistic and antagonistic interactions between pathogens [4],[5].

The human nasopharynx is considered the niche from which respiratory tract infections originate [6]. Several residents of the nasopharyngeal microbiome, including *Streptococcus pneumoniae*, *Haemophilus influenzae*, *Moraxella catarrhalis* and *Staphylococcus aureus*, are major contributors to disease in childhood. However, they are also common, transient colonizers of the nasopharynx of healthy children, especially in the youngest, whose immune systems are still maturing. Colonization of this niche in the upper respiratory tract appears to be a dynamic process of acquisition and elimination of various microbes, during which they interact with the host, its maturing immune system and each other [6]. In a balanced state, this bacterial ecosystem is assumed to be beneficial for health, for example by stimulating the immune system and functioning as a protective barrier against invading pathogens [7].

Viruses can also be frequently detected in nasopharyngeal samples of healthy children [8]–[10]. Episodes of new bacterial or viral acquisition potentially disturb the equilibrium of this ecosystem, upon which pathogens could have an opportunity to invade, disseminate and cause diseases like acute otitis media, pneumonia or even meningitis [4]. Importantly, dynamics in exposure to viruses and bacteria are influenced by environmental factors, like crowding at day care or siblings living in the same household [6]. Knowledge about the prevalence of bacteria and viruses in the nasopharynx of healthy children as well as specific associations between these pathogens is important to interpret findings during disease and to ultimately better understand pathogenesis of respiratory infections. However, this is still a rather unexplored field of research.

Here, we describe the results of a post-hoc analysis in 986 nasopharyngeal swab samples from healthy 6- to 24-month-old children who had participated in a pneumococcal vaccination trial. We aimed to evaluate the prevalence of a wide range of common respiratory viruses as well as co-occurrence patterns with four of the most commonly detected bacterial pathogens in clinical practice (*S. pneumoniae*, *H. influenzae*, *M. catarrhalis* and *S. aureus*), while taking well described epidemiologic and environmental determinants into account.

## Materials and Methods

### Ethics Statement

An acknowledged Independent Ethics Committee from the Netherlands (Stichting Therapeutische Evaluatie Geneesmiddelen) approved the study protocol. The trial was undertaken in accordance with the European Statements for Good Clinical Practice, which include the provisions of the Declaration of Helsinki. Written informed consent was obtained from each subject’s parent(s) or legal guardian(s) before enrolment.

### Study Population and Design

We selected 986 nasopharyngeal samples obtained from children who had participated in a randomized controlled trial (ClinicalTrials.gov Identifier NCT00189020)[11]. This trial, designed to assess the effects of reduced-dose schedules of 7-valent pneumococcal conjugate vaccine (PCV-7) on nasopharyngeal pneumococcal colonization, was conducted in the Netherlands between July 2005 and February 2008. Details of the trial and bacterial carriage rates have been reported previously [11]–[13]. In short, a total of 1003 healthy infants were enrolled and randomly assigned to receive either (1) PCV-7 at 2 and 4 months of age, (2) PCV-7 at 2, 4 and 11 months of age, or (3) no dosage (control group). Children were visited at home to obtain nasopharyngeal samples at the age of 6 weeks, 6, 12, 18 and 24 months. Children were generally healthy at the time they were visited–i.e., visits were postponed when parents deemed their child unfit for the study procedures, for example in case of fever or acute symptoms of an infection. At each visit, a questionnaire collecting information on day care attendance, the presence of siblings, and administration of antibiotics was obtained from the parents.

For the present study, nasopharyngeal samples were selected from children in the unvaccinated control group and the 2+1-dose schedule group based on availability of sufficient quantity of remaining materials. Ultimately, 497 samples from the 2+1-dose schedule group and 489 samples from the control group were analyzed. These 986 samples were taken from a total of 433 children: 212 were paired samples from the same children at 6 and 18 months of age, 121 were paired samples from the same children at 12 and 24 months of age, samples from 74 children were collected at four consecutive time points, while from another 26 children only one sample was used.

### Nasopharyngeal Samples and Laboratory Procedures

Nasopharyngeal samples were taken transnasally with a flexible, sterile swab (Transnasal Pernasal Plain, Medical Wire and Equipment Co, Corsham Wiltshire, England) by trained study staff according to World Health Organization standard procedures [14]. Culture and bacterial identification occurred according to standard procedures, as previously described in detail [11]–[13]. After plating, the swabs were rinsed in 1 mL of saline and stored at −80°C until further analysis.

Nucleic acids were extracted from one aliquot of 200 µL swab ‘rinse’ solution using the MagNa pure LC total nucleic isolation (Roche Diagnostics, Basel, Switzerland), as previously described [15]. Samples were tested using real-time PCR specific for human bocavirus, polyomaviruses (WU and KI), respiratory syncytial virus (A and B), human influenza virus A and B, parainfluenza virus 1–4, human rhinoviruses, adenoviruses, human coronavirus OC43, NL63, HKU and 229E, human metapneumovirus, human parechovirus, and enteroviruses. Primers, probes and PCR assay conditions used for this study have been previously reported in detail [15]–[17]. The presence of human parechovirus and enteroviruses was determined in a subgroup of 831 samples due to limited amounts of nucleic acids that had remained available to run these final tests.

### Statistical Analysis

The bacterial colonization rate was defined as the proportion of nasopharyngeal samples positive for a particular bacterium by conventional culture. Likewise, the viral detection rate was defined as the proportion of samples positive for a particular virus by real-time PCR. For convenience of statistical analyses, cycle threshold (Ct) values were dichotomized and the different subtypes of viruses belonging to a specific group (e.g., parainfluenza viruses) were pooled. Ct values <45 were defined as positive, i.e., if the sample did not become positive after 45 cycles, viruses assayed for were defined to not be present.

First, we explored univariate associations amongst the four bacteria and between each bacterium and risk factors for bacterial colonization–i.e., age, presence of siblings, day care attendance, recent antibiotic use (i.e., within two months before sampling), and vaccination with PCV-7. We calculated the risk and 95% confidence interval (CI) for each bacterium to co-occur with another bacterium (or risk factor) relative to the presence of that bacterium in absence of another bacterium (or risk factor). Similarly, we assessed the co-occurrence of each of the bacteria with a particular virus (or pooled group of subtypes). Again, we calculated the risk and 95% CI for each of the bacteria to be present when a particular virus (or pooled group of subtypes) co-occurred relative to the presence of each bacterium in absence of that virus.

Next, we used logistic regression models to examine independent associations between the isolation of bacteria, detection of viruses, and risk factors. All associations with a P value of <0.1 in univariate regression analysis were subsequently entered in multivariate regression models. We verified the age-related distribution of each of the covariates included in the various models, which turned out to be linear. Age was therefore entered into the model as continuous variable. Adjusted odds ratios with their 95% CI’s were computed. In order to retain sufficient statistical power in models that included enterovirus and human parechovirus, missing values were imputed by the single imputation procedure, which suffices in case the number of missing values is limited as in our study [18]. We verified our primary analyses using a repeated measurements model taking more than one measurement per child into account using generalized linear models with an autoregressive correlation structure [19]. Results were virtually the same, indicating that potential within-person dependency was not substantially affecting the precision of our estimates. Results from the primary analyses are presented here. All these analyses were performed with SPSS Statistics version 17.0.

Finally, all variables for which statistically significant associations were found in multivariate analysis were subsequently entered into a single partial correlation matrix. This analysis identifies all independent correlations between any two given parameters in the matrix after correcting for the remaining variables with P-values of 0.01 and 0.05 used as cut-off. This analysis was performed in software package R 2.7 (function cor2por [package for R; available at: http://cran.r-project.org/web/packages/corpcor/index.html]) and visualized using the complex network analysis in Cytoscape (version 2.7) [20]. Rather than correcting for multiple comparisons, correlations at different levels of significance are visualized. Still, significant results should be interpreted with caution.

## Results

Characteristics of the children, nasopharyngeal bacterial colonization and viral detection rates are shown in Table 1. Bacterial colonization rates in the present study were similar to results of the main trial [11]–[13]. Respiratory viruses were detected in almost 70% of samples, with 29% of samples showing evidence of multiple viruses. Human rhinoviruses were detected most frequently, ranging from 31% to 50% of samples (Table 1).

**Table 1:** Characteristics of the children, nasopharyngeal bacterial colonization and viral detection rates.

Characteristic	6-month-old children	12-month-old children	18-month-old children	24-month-old children	Total
	No. (%)	No. (%)	No. (%)	No. (%)	No. (%)
Number of samples (children)	288	198	298	202	986
Age, months (SD)	6.1 (0.39)	12.1 (0.41)	18.1 (0.35)	24.3 (0.69)	NA
Period of sampling					NA
From:	November 2005	October 2006	December 2006	September 2007	
To;	June 2006	January 2007	July 2007	January 2008	
Male sex	162 (56)	105 (53)	162 (54)	107 (52)	536 (54)
PCV-7 vaccination	146 (51)	104 (53)	149 (50)	98 (49)	497 (50)
Day care attendance^a^	159 (55)	127 (64)	186 (62)	149 (74)	621 (63)
Presence of siblings	142 (49)	106 (54)	157 (53)	130 (64)	535 (54)
Recent antibiotic use^b^	22 (8)	23 (12)	35 (12)	11 (5)	91 (9)
Symptoms of URTI^c^	71 (25)	79 (40)	102 (34)	76 (38)	328 (33)
**Bacterial colonization**					
Any bacterium	250 (87)	185 (93)	278 (93)	187 (93)	900 (91)
Multiple bacteria	173 (60)	150 (76)	204 (69)	142 (70)	639 (65)
*S. pneumoniae*	143 (50)	139 (70)	191 (64)	134 (66)	607 (62)
*H. influenzae*	99 (34)	103 (52)	179 (60)	115 (57)	496 (50)
*M. catarrhalis*	190 (66)	158 (80)	215 (72)	148 (73)	711(72)
*S. aureus*	47 (16)	13 (7)	14 (5)	11 (5)	85 (9)
**Viral detection rates**					
Any virus	168 (58)	146 (74)	209 (70)	140 (69)	663 (67)
Multiple viruses	53 (18)	76 (38)	91 (31)	65 (32)	285 (29)
Human rhinovirus	88 (31)	98 (50)	104 (35)	80 (40)	370 (38)
Enterovirus^d^	6 (2)	42 (21)	37 (26)	43 (21)	128 (15)
Human bocavirus	26 (9)	17 (9)	53 (18)	27 (13)	123 (12)
Polyomaviruses (pooled)	30 (10)	33 (17)	43 (14)	41 (20)	147 (15)
WU	16 (6)	28 (14)	34 (11)	33 (16)	111 (11)
KI	14 (5)	5 (3)	9 (3)	8 (4)	36 (4)
Human coronaviruses (pooled)	23 (8)	6 (3)	31 (10)	20 (10)	80 (8)
OC43	6 (2)	5 (3)	22 (7)	0 (0)	33 (3)
NL63	6 (2)	1 (1)	6 (2)	3 (1)	16 (2)
HKU	5 (2)	0 (0)	3 (1)	7 (3)	15 (2)
229E	2 (1)	0 (0)	0 (0)	5 (2)	7 (1)
Unknown	4 (1)	0 (0)	0 (0)	5 (2)	9 (1)
Parainfluenza viruses (pooled)	14 (5)	10 (5)	31 (10)	7 (4)	62 (6)
Type 1	9 (3)	0 (0)	20 (7)	1 (0)	30 (3)
Type 2	1 (0)	0 (0)	0 (0)	2 (1)	3 (0)
Type 3	3 (1)	1 (1)	6 (2)	0 (0)	10 (1)
Type 4	0 (0)	9 (5)	5 (2)	3 (1)	17 (2)
Unknown	1 (0)	0 (0)	0 (0)	1 (0)	2 (0)
Human adenovirus	15 (5)	11 (6)	23 (8)	9 (5)	58 (6)
Human parechovirus^d^	14 (5)	29 (15)	9 (6)	19 (9)	71 (9)
Respiratory syncytial virus	6 (2)	5 (3)	9 (3)	5 (3)	25 (3)
Influenza virus	11 (4)	2 (1)	3 (1)	1 (1)	17 (2)
Human metapneumovirus	2 (1)	1 (1)	1 (0)	0 (0)	4 (0)

Abbreviations: SD, standard deviation; NA, not applicable; PCV-7, 7-valent pneumococcal conjugate vaccine; URTI, upper respiratory tract infection.

a Defined as more than 4 hours per week with at least one child from another family (yes/no).

b Defined as use of an antibiotic, orally or intravenously administered with start date within 2 months before sampling date (yes/no). Of those, the prescribed antibiotic was amoxicillin (n = 69), penicillin (n = 1), amoxicillin/clavulanic acid (n = 3), a macrolide (n = 14; claritromycin (n = 8), azitromycin (n = 5), erythromycin (n = 1), a cephalosporin (n = 1, unknown type), and 3 unknowns.

c Parent-reported presence of mild symptoms of an upper respiratory tract infection (eg, a runny nose) at the time of sampling (yes/no).

d Presence of enteroviruses and human parechovirus was determined in a subgroup of samples (N = 831) due to insufficient amounts of remaining nasopharyngeal swab material or nucleic acids to run these tests. Missing values were imputed by the single imputation procedure in multivariate analysis models in which these viruses were included to retain statistical power.

Results of univariate analyses are shown in detail in the supporting information (Figures S1 and S2). To summarize, *S. pneumoniae, H. influenzae* and *M. catarrhalis* were positively associated with each other (Figure S1A–C), whereas each of them was negatively associated with *S. aureus* (Figure S1D). Day care attendance and presence of siblings in the household were associated with an increased risk of colonization with all bacteria except *S. aureus* (Figure S1). Recent use of antibiotics was associated with a significant decreased risk of pneumococcal colonization as was PCV-7 vaccination, which corresponds to previously described results [11]. In general, *S. pneumoniae*, *H. influenzae*, and *M. catarrhalis* were more likely to be present in the nasopharynx in combination with (multiple) respiratory viruses (Figure S2A–C).

In multivariate analyses, we observed persistent positive associations between *S. pneumoniae* colonization and the presence of *H. influenzae* and *M. catarrhalis*, the presence of siblings, day care attendance, human rhinoviruses, and enteroviruses (Table 2). *S. aureus* carriage, recent antibiotic use, and PCV-7 vaccination remained inversely related with pneumococcal colonization in multivariate analysis. We found no major differences between the risk of co-occurrence of the most prevalent respiratory viruses with pneumococcal vaccine or non-vaccine serotypes. Likewise, we found no differences upon stratification of the analyses for vaccination status. *H. influenzae* colonization was positively associated with the presence of *S. pneumoniae*, human rhinoviruses and respiratory syncytial viruses, the presence of siblings, and day care attendance. *M. catarrhalis* colonization remained positively correlated with the presence of *S. pneumoniae*, coronaviruses, adenoviruses and the presence of siblings. A negative association was found between *M. catarrhalis* and *S. aureus*, with an adjusted odds ratio being even more profound than that between *S. aureus* and *S. pneumoniae* colonization (0.42 *vs* 0.56, respectively). The positive association between the presence of *S. aureus* and the pooled group of influenza viruses persisted in the multivariate model (Table 2).

**Table 2:** Distribution and adjusted odds ratiosa,b for nasopharyngeal bacterial colonization, co-occurrence with each of the other bacteria, respiratory viruses and risk factors.

	*S. pneumoniae*			*H. influenzae*			*M. catarrhalis*			*S. aureus*		
Covariate	Without covariate	With covariate	aOR (95% CI)	Without covariate	With covariate	aOR (95% CI)	Without covariate	With covariate	aOR (95% CI)	Without covariate	With covariate	aOR (95% CI)
	No. (%)	No. (%)		No. (%)	No. (%)		No. (%)	No. (%)		No. (%)	No. (%)	
*S. pneumoniae*		NA		139 (37)	357 (59)	**1.54 (1.15–2.06)**	226 (60)	485 (80)	**1.73 (1.26–2.38)**	51 (13)	34 (6)	**0.56 (0.34–0.91)**
*H. influenzae*	250 (51)	357 (72)	**1.60 (1.18–2.16)**		NA		318 (65)	393 (79)	1.24 (0.89–1.72)	57 (12)	28 (6)	0.72 (0.44–1.21)
*M. catarrhalis*	122 (44)	485 (68)	**1.78 (1.29–2.47)**	103 (37)	393 (55)	**1.27 (0.91–1.76)**		NA		45 (16)	40 (6)	**0.42 (0.25–0.68)**
*S. aureus*	573 (64)	34 (40)	**0.59 (0.35–0.98)**	468 (52)	28 (33)	0.72 (0.43–1.23)	671 (74)	40 (47)	**0.42 (0.25–0.69)**		NA	
Antibiotic use	574 (64)	33 (36)	**0.24 (0.15–0.40)**	450 (50)	46 (51)	NA	643 (72)	68 (75)	NA	75 (8)	10 (11)	NA
Presence of siblings	236 (52)	371 (69)	**2.31 (1.69–3.15)**	181 (40)	315 (59)	**2.42 (1.80–3.26)**	309 (69)	402 (75)	**1.59 (1.14–2.22)**	46 (10)	39 (7)	NA
Day care attendance	183 (50)	424 (68)	**1.70 (1.23–2.36)**	130 (36)	366 (59)	**2.52 (1.83–3.46)**	202 (55)	509 (82)	**3.22 (2.30–4.51)**	47 (13)	38 (6)	0.74 (0.45–1.21)
PCV-7 vaccination	326 (65)	281 (58)	**0.68 (0.51–0.91)**	248 (50)	248 (51)	NA	372 (75)	339 (70)	NA	40 (8)	45 (9)	NA
Human rhinovirus	335 (54)	272 (74)	**1.63 (1.19–2.22)**	267 (43)	229 (62)	**1.63 (1.22–2.18)**	420 (68)	291 (79)	1.21 (0.86–1.69)	54 (9)	31 (8)	NA
Enterovirus	471 (58)	136 (79)	**1.97 (1.26–3.10)**	382 (47)	114 (66)	1.25 (0.84–1.85)	568 (70)	143 (83)	1.27 (0.79–2.05)	74 (9)	11 (6)	NA
Human bocavirus	527 (61)	80 (65)	NA	424 (49)	72 (59)	1.11 (0.73–1.70)	615 (71)	96 (78)	NA	78 (9)	7 (6)	NA
WU polyomavirus	529 (60)	78 (70)	1.32 (0.82–2.14)	430 (49)	66 (59)	1.17 (0.75–1.83)	629 (72)	82 (74)	NA	78 (9)	7 (6)	NA
Human coronavirus	550 (61)	57 (71)	1.38 (0.80–2.38)	456 (50)	40 (50)	NA	642 (71)	69 (86)	**1.99 (1.01–3.93)**	82 (9)	3 (4)	NA
Parainfluenza virus	568 (60)	39 (63)	NA	470 (51)	26 (42)	NA	662 (72)	49 (79)	NA	83 (9)	2 (3)	NA
Adenovirus	561 (60)	46 (79)	1.91 (0.95–3.84)	462 (50)	34 (59)	NA	657 (76)	54 (93)	**3.69 (1.29–10.56)**	83 (9)	2 (3)	NA
Human parechovirus	530 (60)	77 (76)	NA	434 (49)	62 (61)	1.19 (0.75–1.90)	630 (71)	81 (80)	1.17 (0.67–2.05)	76 (9)	9 (9)	NA
KI polyomavirus	586 (62)	21 (58)	NA	482 (50)	14 (39)	NA	683 (72)	28 (78)	NA	84 (9)	1 (3)	NA
RSV	590 (61)	17 (68)	NA	478 (50)	18 (72)	**2.78 (1.06–7.28)**	694 (72)	17 (68)	NA	83 (9)	2 (8)	NA
Influenza virus	596 (62)	11 (65)	NA	487 (50)	9 (53)	NA	701 (72)	10 (59)	NA	79 (8)	6 (35)	**4.87 (1.59–14.89)**

Abbreviations: aOR, adjusted odds ratio; CI, confidence interval; NA, not applicable (i.e., not included in the model for that particular bacterial pathogen), RSV, respiratory syncytial virus.

a Adjusted for age and all variables with a P value of <0.1 in univariate analysis.

b Statistically significant associations are shown in bold.

Multivariate models for independent associations with the most frequently detected viruses are shown in Table 3. Human rhinoviruses were positively associated with the presence of siblings as well as with enteroviruses, and negatively associated with coronaviruses. In addition, enteroviruses were positively associated with the presence of human bocavirus, WU polyomavirus, parainfluenza viruses and human parechovirus. Human bocavirus was also associated with day care attendance and the presence of WU polyomavirus (Table 2).

**Table 3:** Distribution and adjusted odds ratiosa,b for nasopharyngeal presence of the most common viruses, co-occurrence with each of the other respiratory viruses, bacteria and risk factors.

	Human rhinovirus			Enterovirus			Human bocavirus			WU polyomavirus		
Covariate	Without covariate	With covariate	aOR (95% CI)	Without covariate	With covariate	aOR (95% CI)	Without covariate	With covariate	aOR (95% CI)	Without covariate	With covariate	aOR (95% CI)
	No. (%)	No. (%)		No. (%)	No. (%)		No. (%)	No. (%)		No. (%)	No. (%)	
*S. pneumoniae*	98 (26)	272 (45)	**1.70 (1.25–2.31)**	36 (9)	136 (22)	**1.64 (1.05–2.54)**	43 (11)	80 (13)	NA	33 (9)	78 (13)	1.30 (0.82–2.06)
*H. influenzae*	141 (29)	229 (46)	**1.52 (1.14–2.03)**	58 (12)	114 (23)	1.26 (0.85–1.87)	51 (10)	72 (15)	1.07 (0.71–1.63)	45 (9)	66 (13)	1.13 (0.73–1.75)
*M. catarrhalis*	79 (29)	291 (41)	1.22 (088–1.70)	29 (11)	143 (20)	1.30 (0.80–2.11)	27 (10)	96 (14)	NA	29 (11)	82 (12)	NA
*S. aureus*	339 (38)	31 (36)	NA	161 (18)	11 (13)	NA	116 (13)	7 (8)	NA	104 (12)	7 (8)	NA
Antibiotic use	343 (38)	27 (30)	NA	153 (17)	19 (21)	NA	107 (12)	16 (3)	NA	101 (12)	10 (11)	NA
Presence of siblings	146 (32)	224 (42)	**1.41 (1.05–1.90)**	75 (17)	97 (18)	NA	62 (14)	61 (11)	NA	51 (11)	60 (11)	NA
Day care attendance	108 (30)	262 (42)	1.37 (0.99–1.89)	34 (9)	138 (22)	1.56 (0.98–2.49)	25 (7)	98 (16)	**2.04 (1.26–3.31)**	32 (9)	79 (13)	1.07 (0.67–1.71)
PCV-7 vaccination	191 (38)	179 (37)	NA	79 (16)	93 (19)	NA	59 (12)	64 (13)	NA	47 (9)	64 (13)	1.46 (0.97–2.20)
Human rhinovirus		NA		69 (11)	103 (28)	**2.50 (1.72–3.63)**	71 (12)	52 (14)	NA	61 (10)	50 (14)	1.16 (0.76–1.78)
Enterovirus	267 (33)	103 (60)	**2.40 (1.66–3.47)**		NA		78 (10)	45 (26)	**2.48 (1.59–3.88)**	75 (9)	36 (21)	**1.71 (1.05–2.78)**
Human bocavirus	318 (37)	52 (42)	NA	127 (15)	45 (37)	**2.78 (1.75–4.42)**		NA		82 (10)	29 (24)	**2.38 (1.44–3.91)**
WU polyomavirus	320 (34)	50 (45)	1.11 (0.72–1.72)	136 (16)	36 (32)	**1.70 (1.03–2.80)**	94 (11)	29 (26)	**2.34 (1.42–3.87)**		NA	
Human coronavirus	355 (39)	15 (19)	**0.27 (0.15–0.49)**	153 (17)	19 (24)	NA	115 (13)	8 (10)	NA	101 (11)	10 (13)	NA
Parainfluenza virus	350 (38)	20 (32)	NA	155 (17)	17 (27)	**2.06 (1.06–3.99)**	111 (12)	12 (19)	1.55 (0.77–3.10)	103 (11)	8 (13)	NA
Adenovirus	339 (37)	31 (53)	1.53 (0.86–2.74)	154 (17)	18 (31)	1.76 (0.93–3.33)	115 (12)	8 (14)	NA	106 (11)	5 (9)	NA
Human parechovirus	317 (36)	53 (52)	1.40 (0.89–2.20)	134 (15)	38 (38)	**2.77 (1.69–4.55)**	110 (12)	13 (13)	NA	95 (11)	16 (16)	NA
KI polyomavirus	360 (38)	10 (28)	NA	166 (17)	6 (17)	NA	115 (12)	8 (22)	2.28 (0.96–5.40)	108 (11)	3 (8)	NA
RSV	364 (38)	6 (24)	NA	168 (17)	4 (25)	NA	120 (12)	3 (12)	NA	108 (11)	3 (12)	NA
Influenza virus	367 (38)	3 (18)	NA	170 (18)	2 (12)	NA	122 (13)	1 (6)	NA	110 (11)	1 (6)	NA

Abbreviations: aOR, adjusted odds ratio; CI, confidence interval; NA, not applicable (i.e., not included in the model for that particular virus or pooled group of viruses), RSV, respiratory syncytial virus.

a Adjusted for age and all variables with a P value of <0.1 in univariate analysis.

b Statistically significant associations are shown in bold.

**Figure 1:**
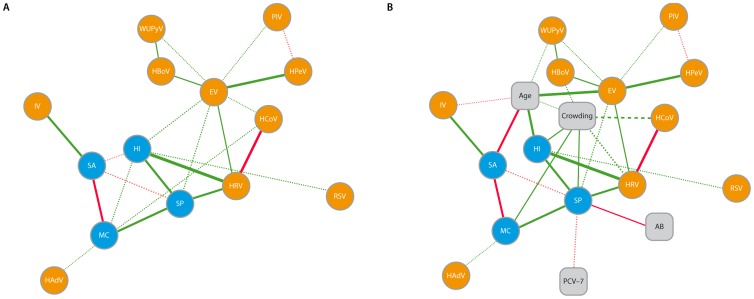
Graphical representation of interaction patterns. Visualization of the partial correlations between bacteria and viruses (A) and epidemiologic drivers (risk factors) of those interactions (B). The patterns depicted here result from partial correlation network analysis and are visualized by Cytoscape. Bacteria are shown in blue, respiratory viruses in orange and risk factors in grey boxes. The solid lines represent associations with a p-value less than 0.01, the dashed lines represent associations with a p-value between 0.01 and 0.05. Green lines indicate positively correlated variables; red lines indicate negative correlations. The thickness of the line indicates the magnitude of the correlation. Abbreviations: SP, *S. pneumoniae*; HI, *H. influenzae*; MC, *M. catarrhalis*; SA, *S. aureus*; HRV, human rhinovirus, EV, enterovirus; HBoV, human bocavirus; WUPyV, WU polyomavirus; HCoV, human coronavirus; PIV, parainfluenza virus; HAdV, human adenovirus; IV, influenza virus; HPeV, human parechovirus; RSV, respiratory syncytial virus; AB, antibiotic use within 2 months before sampling; ‘crowding’ was entered into the model as a variable combining the presence of siblings (yes/no) and day care attendance (yes/no); 0 = no siblings and no day care attendance, 1 = siblings present, but not attending day care, or vice versa, and 2 = siblings present and attending day care.

Figure 1 graphically summarizes the results of partial correlation network analysis. All significant associations shown by multivariate analysis persisted in partial correlation network analysis (Figure 1A). When simultaneously adjusting for driving risk factors (Figure 1B) all correlations remain, except for those between *H. influenzae* and *M. catarrhalis* (P = 0.17), and between *M. catarrhalis* and coronaviruses (P = 0.064).

## Discussion

To our knowledge, this is the first study in which co-occurrence patterns of four potentially pathogenic bacteria and 20 common respiratory viruses in nasopharyngeal samples from otherwise healthy young children are investigated by multivariate and partial correlation network analysis. The latter enables a helicopter-view of possible interrelations between microbes in a complex community, simultaneously correcting for the influence of important epidemiologic and environmental determinants [21]. Even in a healthy state, we found numerous specific associations between viral and bacterial pathogens which could have an important role in local ecosystem dynamics. These findings may guide future studies in pursuing possible underlying mechanisms and their role in pathogenesis of respiratory disease.

The bacterial detection rates in our study are comparable to previous studies in young children [11]–[13]. Also, viral detection rates in our study were comparable to previous reports [9],[10]. The abundant presence of viruses in samples from asymptomatic children raises the question whether a positive result could be regarded as causal in case of respiratory disease symptoms. It seems clear that certain viruses, such as respiratory syncytial viruses and influenza viruses, are capable of causing disease on their own [9]. For the more commonly “carried” viruses (eg, rhinovirus, human bocavirus, WU polyomavirus), it has been suggested that not merely presence but rather a certain viral load is needed above which respiratory symptoms may occur [22]. However, considering both their high detection rates and their associations with bacterial colonization, we feel it becomes even more complex to identify an individual microbe or a certain viral load as single cause of a respiratory tract infection in young children.

In general, the risk of *S. pneumoniae*, *H. influenzae* and *M. catarrhalis* colonization seemed to increase in the presence of particular respiratory viruses. The magnitude of this relative risk differed per virus and per bacterium. The presence of influenza viruses in general was associated with an increased risk for colonization by *S. aureus* as well as *S. pneumoniae*, both of which are also supported by observations during flu pandemics. While H1N1 influenza A was associated with *S. aureus* pneumonia in the 2009 pandemic, an association with pneumococcus prevailed during others, depending on the subtype of virus [5],[23]. In the present study, only a positive association between influenza viruses and *S. aureus* persisted in multivariate analysis, which could be a reflection of differential effects of various influenza virus subtypes on these bacterial pathogens [24].

The previously reported negative association between *S. pneumoniae* and *S. aureus* during nasopharyngeal carriage was confirmed in the present study [12],[25],[26]. However, we found an even stronger negative association between *M. catarrhalis* and *S. aureus*. Whether this reflects true microbial interference or, for instance, results from an indirect effect through other (commensal) bacteria or immune modulation warrants further investigation.

Partial correlation analysis provides a two-dimensional view compared to the unidimensional view of classical multivariate analysis [27]. As such, it does not put more value to a single variable over others, and allows for independent associations tested between all parameters simultaneously.

The observed associations are, however, mathematical and need further investigation to unravel their underlying biological mechanisms and to determine whether they are direct or dependent on other (unknown) determinants. Nevertheless, several of the observed statistical associations are supported by an overwhelming body of evidence indicating virus-mediated susceptibility for bacterial infection in the respiratory tract. For instance, research on animal models as well as *in vitro* studies provide biological clues to the positive association between rhinoviruses and *S. pneumoniae* [28],[29]. Of interest was a recently described temporal association between these microbes in disease [30]. This adds to a previously demonstrated association between circulation of influenza and respiratory syncytial viruses, and invasive pneumococcal and meningococcal disease [31].

It is of great interest to gain more insight into the synergism and competition among members of microbial communities of the upper respiratory tract to better understand progression towards disease. A balance in its polymicrobial composition and diversity is assumed to be important for maintenance of a healthy state [4],[6]. For example, it has previously been shown that *S. pneumoniae* can kill *S. aureus* by remote-control bacteriophage induction, possibly accounting for the negative association between those species observed in carriage studies [32]. Research on microbiota of the intestinal tract has made clear that symbiotic bacteria are co-dependent because of shared metabolic pathways [33]. Possibly, a similar mechanism by which bacteria interfere with each other’s presence could also be at play in the upper respiratory tract. We should also note the enormous heterogeneity of highly adaptive bacteria such as *S. pneumoniae* and *H. influenzae* in this context. Although the observed positive association in our study has been previously reported [34], antagonistic effects have also been described to occur [35].

With respect to respiratory disease, it has been shown that pneumococcal conjugate vaccination also reduced virus-associated pneumonia in general, and cases associated with seasonal viruses such as influenza and parainfluenza viruses in particular [36]. This suggests that presumed ‘virus-associated pneumonia’ is actually polymicrobial in nature. The same may be true for acute otitis media, a disease that can be caused by individual pathogens but could also be an end stage of true polymicrobial pathogenesis. In either case, it appears that the old paradigm on viruses predisposing to secondary bacterial disease is an oversimplification of the complexity and dynamics of potential interactions. For example, interdependence between viruses and bacteria occurring in the gut was recently described: some viruses cover themselves with molecules from bacterial residents to make a viral infection possible [37],[38]. This also contradicts the predominant view that resident microbes merely protect against new viral infection.

Some limitations need to be mentioned. First, we modeled the results of the current standard for detecting bacteria (conventional culture) with that for detecting viruses (qPCR). Use of PCR instead of conventional culture may have increased the detection of (less abundant) bacteria. We restricted analysis to four culturable bacterial pathogens, since they are generally considered to be the major contributors to respiratory disease in childhood. Additionally, since bacteria need to be present in sufficient abundance to be detected by culture, these results may reveal the most clinically relevant associations. Also, the prevalence rates of cultured *S. pneumoniae*, *H. influenzae* and *M. catarrhalis* were already high in our study. We recognize that the microbial communities in the upper respiratory tract are inherently complex and include non-culturable, less abundant commensal bacterial species [39],[40]. How these fit into the picture and relate to pathogenesis is an interesting topic of current and future research. Second, by assessing the prevalence of viruses and bacteria in a cross-sectional manner; we only describe associations and cannot speculate on causality. Future studies are needed to show the importance of these associations and possible underlying mechanismsThird, we focused on the crude presence or absence of viruses and bacteria, ignoring viral and/or bacterial load. Finally, it should be noted that, besides possible interactions with members of the flora not assayed for in our study, the demonstrated associations between microbes could, at least partially, be influenced by host genetics and immune maturation status.

Strengths of our study include the large sample size of healthy children, a wide range of viruses included in the analyses and the availability of detailed data on risk factors. The relatively high bacterial colonization and viral detection rates allowed for solid statistical analyses, summarized as a “network model” of distinct associations between these microbes.

In conclusion, we have demonstrated that bacterial pathogens and respiratory viruses are abundantly present in the upper respiratory tract of otherwise healthy young children. The distinct associations between viruses and bacteria found in our study warrant careful consideration when associating microbial presence with specific respiratory diseases.

## Supporting Information

Figure S1 Bacterial colonization in relation to the co-occurrence with other pathogenic bacteria.
(PDF)

Figure S2 Bacterial colonization in relation to the co-occurrence with respiratory viruses.
(PDF)
