# Supplementary material for: Associations between Pathogens in the Upper Respiratory Tract of Young Children: Interplay between Viruses and Bacteria
Source: PLoS One. 2012 Oct 17;7(10):e47711. doi: 10.1371/journal.pone.0047711 (PMC3474735; doi:10.1371/journal.pone.0047711)
Supplement: Figure S1 — Bacterial colonization in relation to the co-occurrence with other pathogenic bacteria. (PDF) [file pone.0047711.s001.pdf]

Figure S1. Bacterial colonization in relation to co-occurrence with other pathogenic bacteria

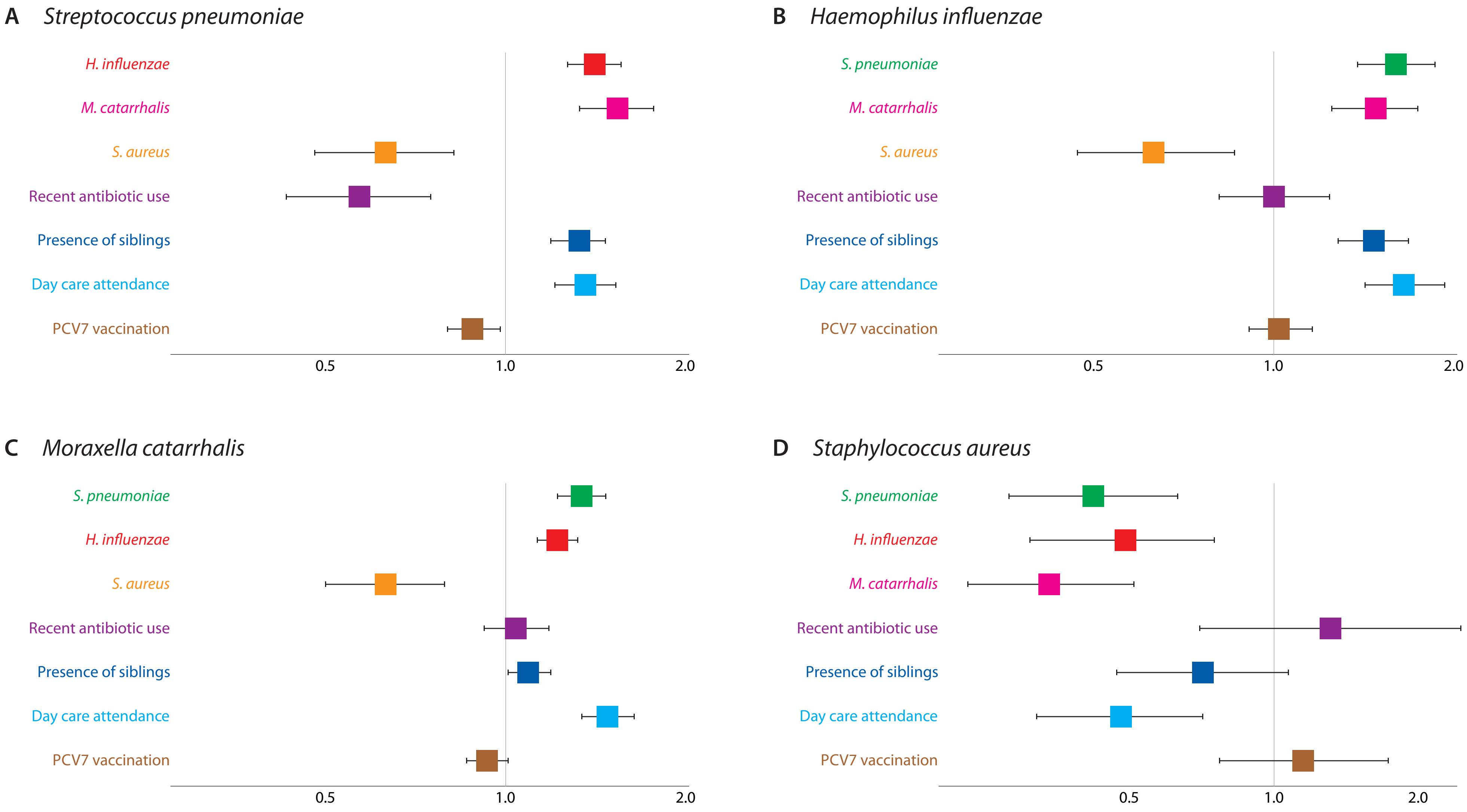

Point estimates are shown for the risk of nasopharyngeal colonization of [A] *S. pneumoniae*, [B] *H. influenzae*, [C] *M. catarrhalis* and [D] *S. aureus* in the presence of another bacterium (or risk factor) relative to colonization in the absence of that particular bacterium (or risk factor). Relative risks are plotted on a logarithmic scale. The horizontal bars indicate 95% confidence intervals.
