# Supplementary material for: Associations between Pathogens in the Upper Respiratory Tract of Young Children: Interplay between Viruses and Bacteria
Source: PLoS One. 2012 Oct 17;7(10):e47711. doi: 10.1371/journal.pone.0047711 (PMC3474735; doi:10.1371/journal.pone.0047711)
Supplement: Figure S2 — Bacterial colonization in relation to the co-occurrence with respiratory viruses. (PDF) [file pone.0047711.s002.pdf]

Figure S2. Bacterial colonization in relation to the co-occurrence with respiratory viruses

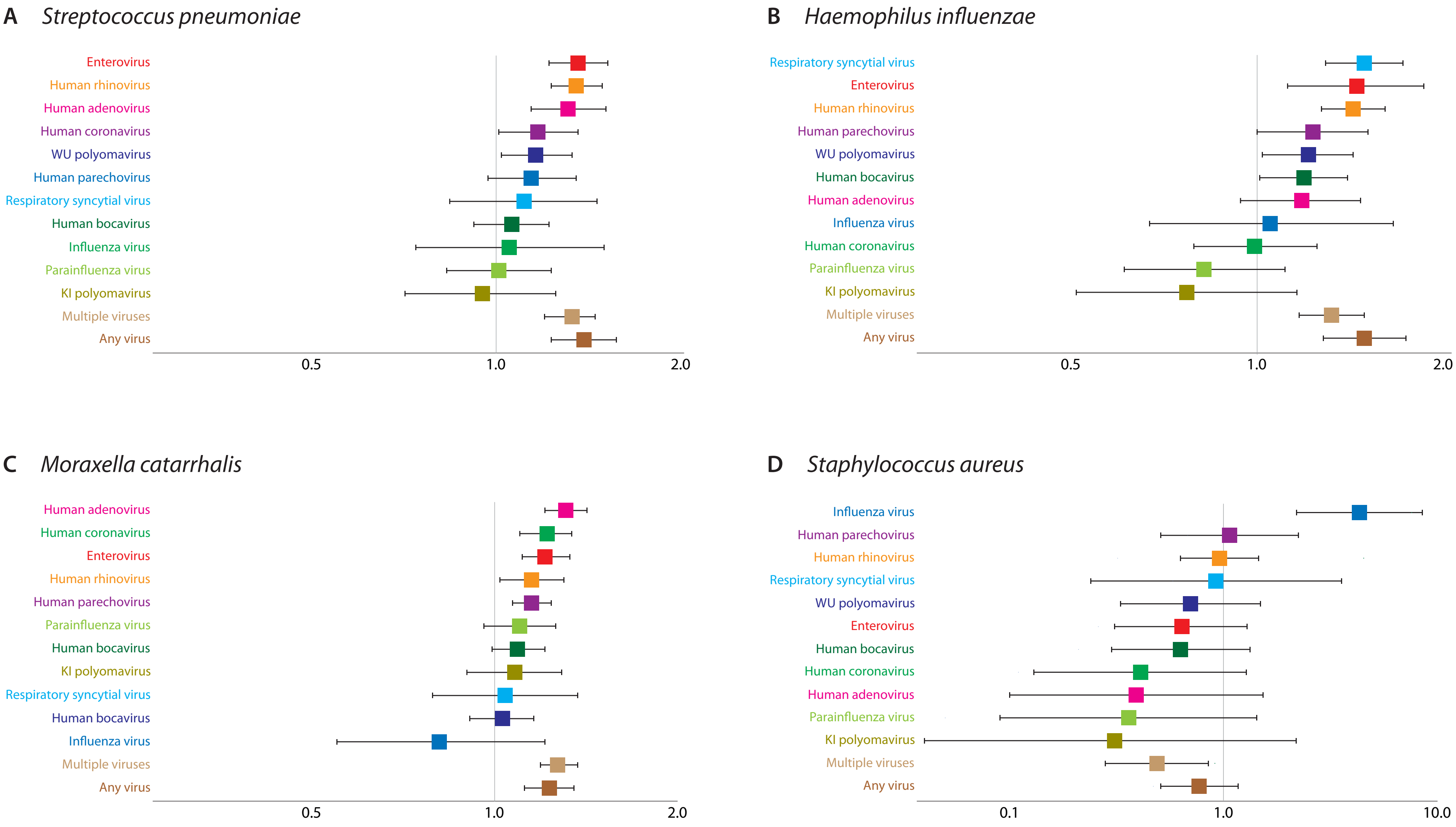

Point estimates are shown for the risk of nasopharyngeal colonization of [A] *S. pneumoniae*, [B] *H. influenzae*, [C] *M. catarrhalis* and [D] *S. aureus* in the presence of a respiratory virus relative to colonization in the absence of that particular virus. Relative risks are plotted on a logarithmic scale. The horizontal bars indicate 95% confidence intervals. *Note that the scale in the figure for S. aureus differs from the others.*
